# A dataset of histograms of original and fake voice recordings (H-Voice)

**DOI:** 10.1016/j.dib.2020.105331

**Published:** 2020-02-26

**Authors:** Dora M. Ballesteros, Yohanna Rodriguez, Diego Renza

**Affiliations:** Universidad Militar Nueva Granada, Colombia

**Keywords:** Fake voice, Machine learning, Convolutional neural networks, Binary classification, Imitation, Deep voice, H-Voice

## Abstract

This paper presents H-Voice, a dataset of 6672 histograms of original and *fake* voice recordings obtained by the Imitation [1,2] and the Deep Voice [3] methods. The dataset is organized into six directories: *Training_fake*, *Training_original*, *Validation_fake*, *Validation_original*, *External_test1*, and *External_test2*. The training directories include 2088 histograms of *fake* voice recordings and 2020 histograms of original voice recordings. Each validation directory has 864 histograms obtained from *fake* voice recordings and original voice recordings. Finally, External_test1 has 760 histograms (380 from *fake* voice recordings obtained by the Imitation method and 380 from original voice recordings), and External_test2 has 76 histograms (72 from *fake* voice recordings obtained by the Deep Voice method and 4 from original voice recordings). With this dataset, the researchers can train, cross-validate and test classification models using machine learning techniques to identify *fake* voice recordings.

**Table:** Specifications Table

Subject	Computer Vision and Pattern Recognition
Specific subject area	Image processing related to identify/classify tampered data
Type of data	Images
How data were acquired	The images were obtained by calculating the histogram of original and fake voice recordings from a repository of the Deep Voice (https://audiodemos.github.io/) and the Imitation methods (https://doi.org/10.17632/ytkv9w92t6.1)
Data format	Raw: histograms (PNG)
Parameters for data collection	The voice recordings are re-quantized to 16 bits. The histograms with 2^16^ bins are calculated from the voice recording (original or fake)
Description of data collection	The dataset is composed by six directories, organized as follows: 1. *Training_fake*: 2088 histograms from *fake* voice recordings (by the Imitation and the Deep Voice methods) 2. *Training_original*: 2020 histograms from original voice recordings 3. *Validation_fake:* 864 histograms from *fake* voice recordings (by the Imitation method) 4. *Validation_original*: 864 histograms from original voice recordings 5. *External_test1*: 760 histograms from original and fake voice recordings (by the Imitation method) 6. *External_test2*: 76 histograms from original and fake voice recordings (by the Deep voice method)
Data source location	City: Bogotá Country: Colombia
Data accessibility	Repository name: Mendeley Data name: H-Voice: Fake voice histograms (Imitation + DeepVoice) [4] Direct URL to data: https://doi.org/10.17632/k47yd3m28w.4

**Table:** 

**Value of the Data** • This is the first dataset of histograms from original and *fake* voice recordings. The histograms are obtained from real signals (original and fake) using the Imitation [1,2] and the Deep Voice [3] methods. • This dataset of histograms allows *fake* voice classifiers to be trained, cross-validated and tested using machine learning techniques such as convolutional neural networks, like how it is done in anti-spoofing speaker verification systems that use spectrograms as features [5,6]. • The dataset is balanced between original and *fake* voice recordings which is a desirable condition to obtain a good trade-off between precision and recall. • This dataset can be used for comparing the performance of different *fake* voice classification models.

## 1 Data description

This dataset is composed by histograms (images) from original and *fake* voice recordings obtained by two methods: Imitation [1,2] and Deep Voice [3]. This data set has four versions in Mendeley, the difference between them corresponding to the number of histograms. Version 1 has 3432 histograms, version 2 has 3792 histograms and version 3 has 6672 histograms. In version 4, corrupted images have been fixed. The latest version (i.e. version 4) is the one explained in this document, which is organized in six directories: *Training_original*, *Training_fake*, *Validation_original*, *Validation_fake*, *External_test1*, and *External_test 2* [4].

**Fig. 1:**
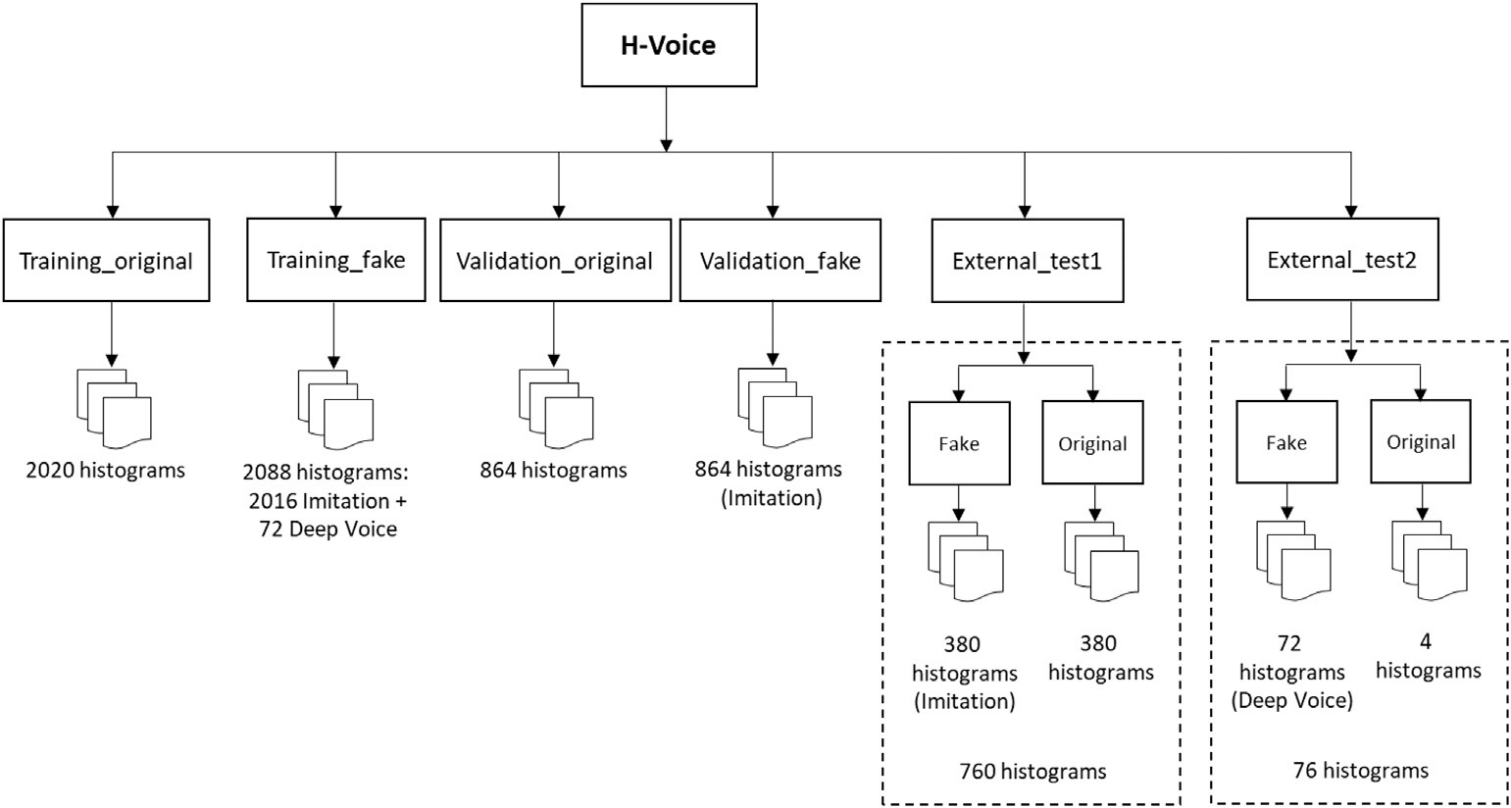
H-Voice dataset structure.

Fig. 1 shows the structure of the dataset. This is explained below:

1. *Training_original*: 2020 histograms from original voice recordings.
2. *Training_fake*: 2088 histograms from *fake* voice recordings, of which 2016 histograms are obtained by the Imitation method, and 72 by the Deep Voice method.
3. *Validation_original*: 864 histograms from original voice recordings.
4. *Validation_fake*: 864 histograms from *fake* voice recordings obtained by the Imitation method.
5. *External_test1*: this is composed of 380 histograms of original voice recordings and 380 histograms of *fake* voice recordings obtained by the Imitation method.
6. *External_test2*: this is composed of 4 histograms of original voice recordings and 72 histograms of *fake* voice recordings obtained by the Deep Voice method.

**Fig. 2:**
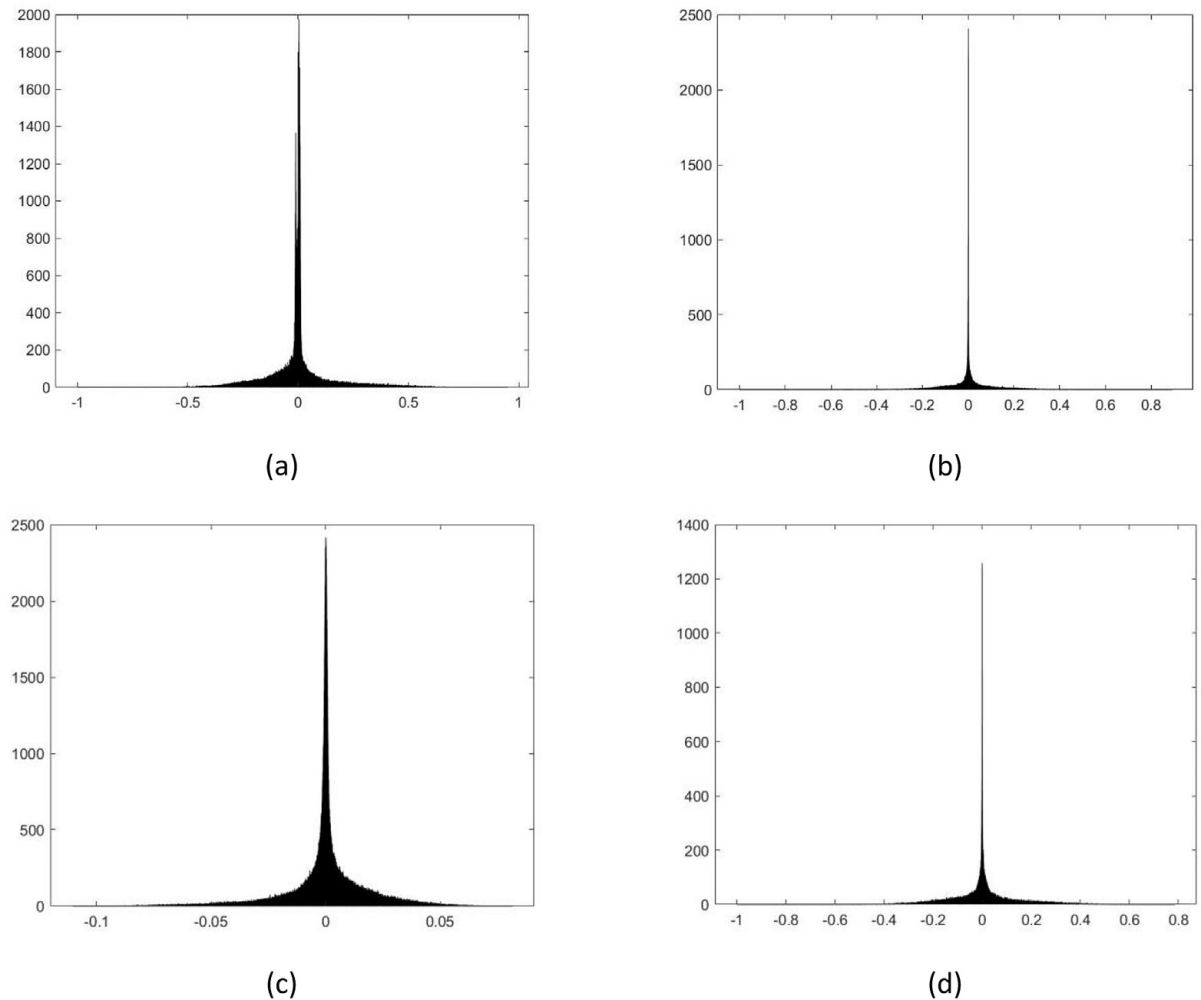
First example of histograms, located at: a)*Training_original*, b)*Training_fake*, c)*Validation_original*, and d)*Validation_fake* directories.

**Fig. 3:**
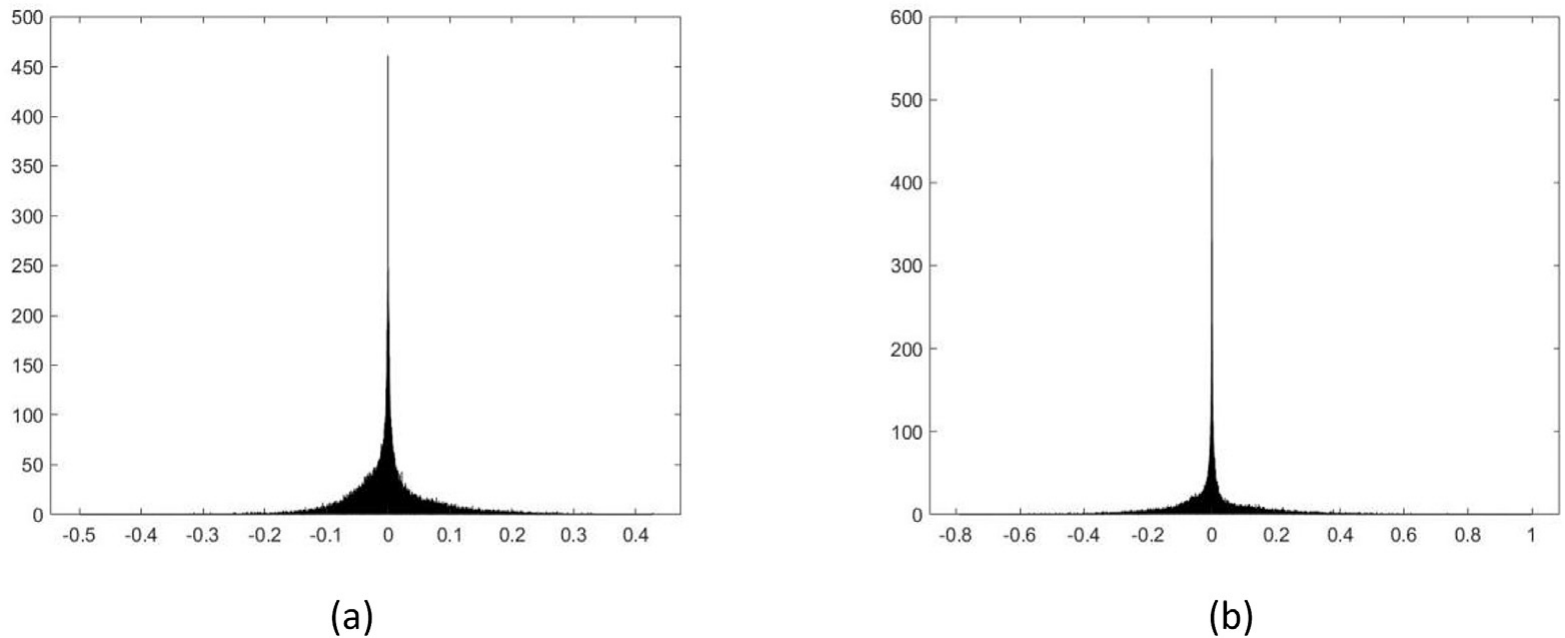
Second example of histograms, located at *External_test1* directory): a) original voice recording, b) *fake* voice recording obtained by the Imitation method.

**Fig. 4:**
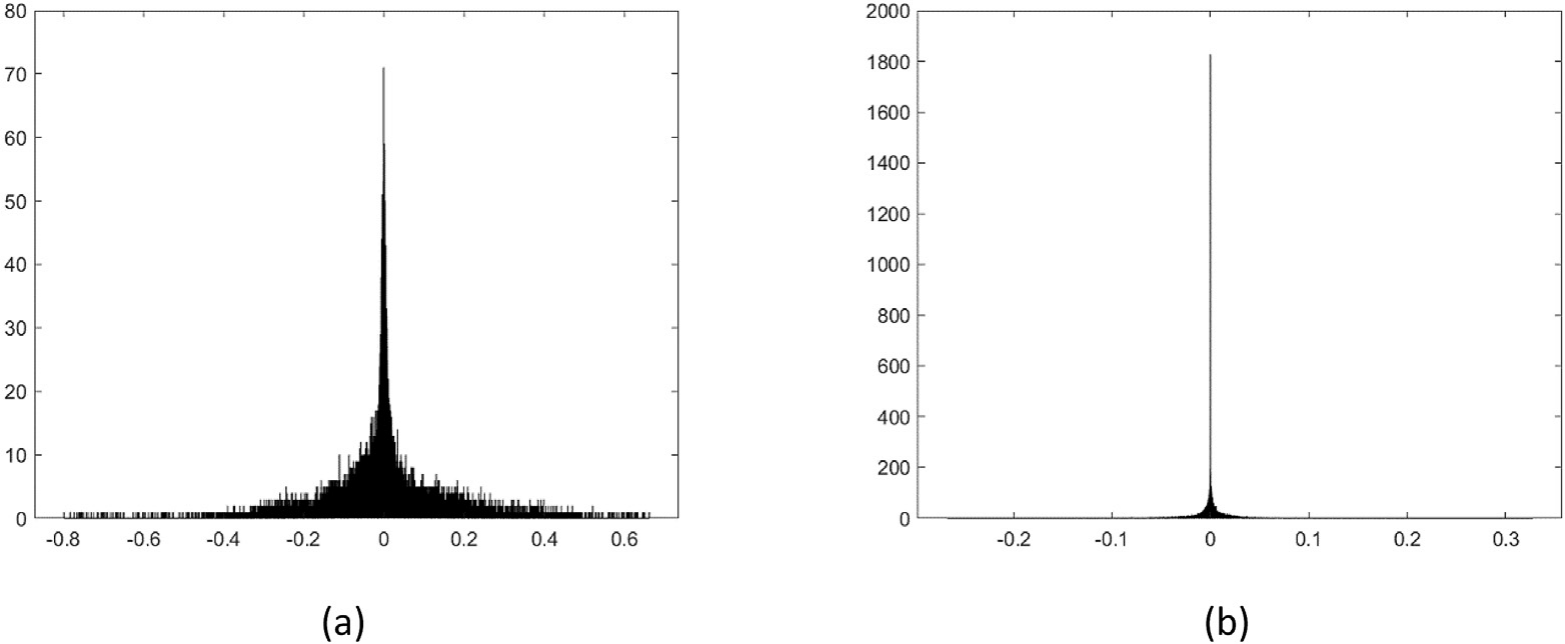
Third example of histograms, located at *External_test2* directory): a) original voice recording, b) *fake* voice recording obtained by the Deep Voice method.

Fig. 2 shows examples of histograms of original and *fake* voice recordings of the training and validation directories. Fig. 3 and Fig. 4 show examples of the *External_test1* and *External_test2* directories, respectively.

## 2 Experimental design, materials, and methods

Fake voice files are created entirely by a machine, either by machine learning (e.g. the Deep Voice method) or by signal processing techniques (e.g. the Imitation method). Unlike *false* voice recordings that are obtained by spoofing the voice, or by manipulating an original voice signal with insertion tasks, deletion or splicing. In the case of the Deep Voice method, a convolutional neural network is trained with original voice recordings to create new (fake) voice recordings with different plain text than the original. On the other hand, the Imitation method uses a re-ordering process of the wavelet coefficients of the original voice signal by imitating the genre, intonation and rhythm of another speaker.

The first step in creating our histograms was to obtain examples of *fake* voice recordings from the Deep Voice and the Imitation methods. In the case of Deep Voice, we use the voice recordings publicly available at https://audiodemos.github.io/. But, in the case of Imitation, we ourselves created *fake* voice recordings with the following code (based on the algorithm proposed in Ref. [2]):

% Inputs: original.wav, target.wav.
% Outputs: fake.wav, key.
[original, FS] = audioread(‘original.wav’); % read the original voice recording.
[target, FS] = audioread(‘target.wav’); % read the target voice recording (to be imitated).
[C1,L1] = wavedec(target,4,‘db10’); % obtain the wavelet coefficients of the original voice recording.
[C2,L2] = wavedec(original,4,‘db10’); % obtain the wavelet coefficients of the target voice recording.
[B1,IX1] = sort(C1,‘descend’); % sort the wavelet coefficients of the original voice recording.
[B2,IX2] = sort(C2,‘descend’); % sort the wavelet coefficients of the target voice recording.
C2m(IX1) = C2(IX2); % re-ordering the wavelet coefficients of the original voice recording.
key(IX1) = IX2; % obtain the key to reverse the process.
fake = waverec(C2m,L1,‘db10’); % create the fake voice obtained from the original voice recording.
audiowrite(‘fake.wav’,fake,FS,‘BitsPerSample’,16); % save the fake voice recording.

Examples of original and *fake* voice recordings obtained with the above algorithm are available at https://doi.org/10.17632/ytkv9w92t6.1.

Once the *fake* voice recordings have been generated, the following code in Matlab allows us to draw the histograms (original/fake):

% Input: name.wav.
% Output: histogram of the voice recording.
[voice, FS] = audioread(‘name.wav’); % read the original/fake voice recording.
nbins = 65536; % number of bins of the histogram.
h = histogram(x, nbins); % plot the histogram.

It is important to note that the examples of *fake* voice recordings obtained by Deep Voice published at https://audiodemos.github.io/have been re-quantized to 16-bits before their histograms were obtained.
